# Association between systemic inflammation response index and depression in U.S. adults: A cross-sectional study from NHANES 2005–2016

**DOI:** 10.1097/MD.0000000000050379

**Published:** 2026-08-21

**Authors:** Qiongqiong Zhan, Yu Wu, Jiaojiao Sun, Yunan Liang, Xinfeng Jiang, Fei Ye

**Affiliations:** a Department of Clinical Psychology, Yangzhou Wutaishan Hospital, Yangzhou, Jiangsu, China.

**Keywords:** cross-sectional study, depression, inflammation, NHANES, systemic inflammation response index

## Abstract

Inflammatory dysregulation has been implicated in depression; however, the association between the systemic inflammation response index (SIRI), a composite inflammatory marker derived from peripheral neutrophil, monocyte, and lymphocyte counts, and depression in the general adult population remains unclear. This study aimed to examine the association between SIRI and depression among adults from the National Health and Nutrition Examination Survey 2005 to 2016. This cross-sectional study included 24,188 adults with available data on the Patient Health Questionnaire-9 (PHQ-9), blood cell counts, and relevant covariates. SIRI was calculated as neutrophil count × monocyte count/lymphocyte count. Depression was assessed using the PHQ-9, with a score ≥10 indicating clinically relevant depressive symptoms. Multivariable logistic regression, restricted cubic spline analysis, threshold effect analysis, and receiver operating characteristic curve analysis were performed to evaluate the association, nonlinear relationship, and incremental discriminatory value of SIRI. Among 24,188 participants, 2055 (8.50%) had clinically relevant depressive symptoms. Higher SIRI was associated with increased odds of depression after multivariable adjustment (odds ratio = 1.07, 95% confidence interval: 1.02–1.13, *P* = .004). Compared with the lowest quartile, participants in the highest SIRI quartile had higher odds of depression (odds ratio = 1.26, 95% confidence interval: 1.10–1.44, *P* < .001). Restricted cubic spline analysis demonstrated a significant nonlinear association between SIRI and depression (*P* for nonlinear = .007). Threshold analysis identified an inflection point at approximately 2.58, below which higher SIRI was associated with increased odds of depression, whereas the association plateaued thereafter. Adding SIRI to the basic model slightly improved discrimination (area under the curve: 0.716 vs 0.725). Higher SIRI levels were associated with higher odds of clinically relevant depressive symptoms among adults from the National Health and Nutrition Examination Survey 2005 to 2016, with a nonlinear dose-response pattern. Given the modest effect size and limited improvement in discrimination, SIRI should be interpreted as an auxiliary peripheral inflammatory marker rather than a standalone screening or diagnostic tool. Further prospective studies are required to clarify causality and clinical relevance.

## 1. Introduction

Depression is a major global public health problem. It was estimated that approximately 280 million people worldwide were affected by depression in 2019, and depression has become one of the leading contributors to nonfatal disease burden.^[1]^ Therefore, identifying accessible biomarkers associated with depression is important for understanding its pathophysiology, recognizing high-risk populations, and informing public health interventions.

In recent years, research on the pathophysiology of depression has expanded beyond the traditional monoamine neurotransmitter hypothesis to include neuroendocrine dysfunction, metabolic abnormalities, immune inflammation, and oxidative stress. Among these mechanisms, immune-inflammatory dysregulation is considered an important pathway linking psychosocial stress, somatic diseases, and depression.^[2]^ Previous studies have shown that patients with depression may exhibit elevated proinflammatory cytokines, activation of inflammatory responses, and alterations in peripheral immune cell profiles. A meta-analysis also reported significantly higher peripheral tumor necrosis factor-alpha (TNF-α) and interleukin (IL)-6 levels in patients with depression than in healthy controls.^[3]^ Inflammatory signals may affect neurotransmitter metabolism, synaptic plasticity, and emotion-regulation networks through mechanisms such as the blood-brain barrier, vagal afferent signaling, and the tryptophan-kynurenine pathway.^[4,5]^ In epidemiological studies, inflammatory markers such as C-reactive protein, IL-6, and TNF-α are commonly used to assess inflammatory burden; however, these markers are relatively costly to measure and are not always available in large-scale population studies. In contrast, composite inflammatory indices derived from routine blood cell counts are convenient, inexpensive, and clinically accessible. The systemic inflammation response index (SIRI), calculated as neutrophil count (NC) × monocyte count (MC)/lymphocyte count (LC), integrates information on innate immune activation and adaptive immune status.^[6]^ Recent studies have suggested that SIRI has prognostic or risk-stratification value in cancer, infectious diseases, and other chronic inflammation-related conditions.^[7,8]^

Given the close relationship between depression and immune-inflammatory dysregulation, SIRI may serve as a potential peripheral marker for identifying individuals at risk of depression. Although previous studies have investigated associations between systemic inflammatory indices and depression-related outcomes, most have primarily focused on linear associations or selected populations. Whether the relationship between SIRI and depression follows a nonlinear dose-response pattern, whether clinically relevant threshold effects exist, and whether SIRI provides additional discriminatory information beyond conventional risk factors remain insufficiently explored.^[9,10]^ Therefore, this study aimed to comprehensively characterize the association between SIRI and depression among adults using National Health and Nutrition Examination Survey (NHANES) 2005 to 2016 data by evaluating nonlinear relationships, threshold effects, subgroup heterogeneity, and incremental discriminatory value.

Therefore, using data from the NHANES 2005 to 2016, the present study included adults with available Patient Health Questionnaire-9 (PHQ-9) depression assessment, differential blood cell counts, and major covariates, and applied logistic regression, restricted cubic spline (RCS) analysis, threshold effect analysis, subgroup analysis, and receiver operating characteristic (ROC) curve analysis to systematically evaluate the association between SIRI and depression risk, potential nonlinear dose-response relationships, effect modification across demographic, socioeconomic, lifestyle, and metabolic subgroups, and incremental discriminatory value.

## 2. Methods

### 2.1. Study design and data source

This was a cross-sectional study based on data from the NHANES 2005 to 2016. NHANES is conducted by the National Center for Health Statistics and uses a complex, multistage, stratified probability sampling design to assess the health and nutritional status of the noninstitutionalized U.S. population. NHANES sampling weights, strata, and primary sampling units were incorporated in all analyses to account for the complex survey design and obtain nationally representative estimates. NHANES data are collected through household interviews, physical examinations, and laboratory tests. The present study used publicly available and de-identified data for secondary analysis. This study was reported according to the Strengthening the Reporting of Observational Studies in Epidemiology guideline for cross-sectional studies.

### 2.2. Study population

**Figure 1.**
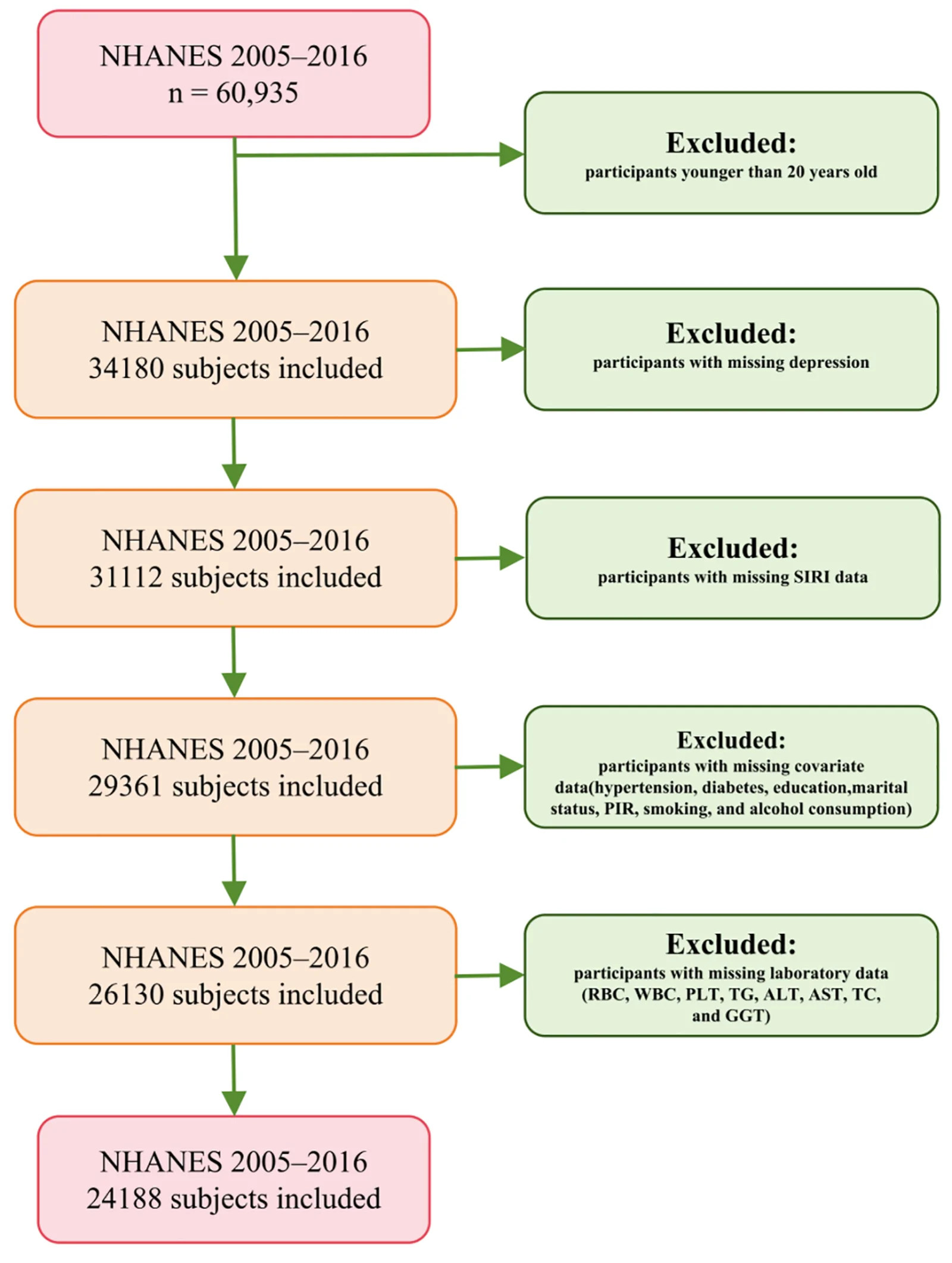
Participant selection flowchart. NHANES = National Health and Nutrition Examination Survey.

Adult participants from NHANES 2005 to 2016 were included in this study. Participants were excluded if they had missing data on depression assessment, missing neutrophil, monocyte, or LCs, or missing information on major covariates. Finally, 24,188 participants were included in the baseline characteristic analysis, of whom 2055 met the criterion for depression and 22,133 did not. For multivariable logistic regression and subgroup analyses, participants with missing covariates in the corresponding models were further excluded, leaving 24,145 participants for regression analyses. The participant selection process is shown in Figure 1, and the number and proportion of participants excluded at each step are summarized in Supplementary Table 1, Supplemental Digital Content 1.

### 2.3. Definition of SIRI

Differential blood cell counts, including neutrophils, monocytes, and LCs, were measured using standardized laboratory methods at the NHANES Mobile Examination Center. The SIRI was used as a composite peripheral inflammatory marker reflecting the relationship between innate immune activation and adaptive immune balance.

SIRI was calculated as follows:


$$\begin{array}{cc} SIRI=neutrophil\;count\\ & \times monocyte\;count/lymphocyte\;count \end{array}$$

That is:


$$\begin{array}{c} \text{S}IRI=NC\times MC/LC \end{array}$$

Where NC represents NC, MC represents MC, and LC represents LC. This calculation was based on the original study that proposed SIRI as a systemic inflammatory index.^[6]^ In the primary analysis, SIRI was included as a continuous variable, and odds ratios (ORs) for depression were calculated per 1-unit increase in the original SIRI value. In addition, SIRI was categorized into quartiles according to its distribution to evaluate the association between different SIRI levels and depression risk.

### 2.4. Assessment of depression

Depression was assessed using the PHQ-9 (see Supplementary Table 2, Supplemental Digital Content 2).^[11]^ The PHQ-9 is a widely used tool for assessing depressive symptoms in epidemiological studies and clinical screening. It consists of 9 items, each scored from 0 to 3 according to the frequency of symptoms over the previous 2 weeks. The total PHQ-9 score ranges from 0 to 27, with higher scores indicating more severe depressive symptoms.

According to previous studies and commonly used screening criteria, depression was defined as a PHQ-9 score ≥10, whereas a PHQ-9 score <10 was defined as no depression. This cutoff is generally considered to have good sensitivity and specificity and is suitable for identifying depression at the population level.

### 2.5. Covariate assessment

Demographic and socioeconomic factors included age, sex, race/ethnicity, education level, marital status, and poverty-to-income ratio (PIR), which may influence both inflammatory status and mental health outcomes. Lifestyle factors included smoking and alcohol consumption, as these behaviors are associated with systemic inflammation and depressive symptoms. Anthropometric and clinical indicators included body mass index (BMI), systolic blood pressure, diastolic blood pressure, and hypertension status, which reflect cardiometabolic health and may be related to both inflammation and depression. Laboratory markers included white blood cell count (WBC), platelet count (PLT), albumin, triglycerides (TG), alanine aminotransferase, aspartate aminotransferase, and gamma-glutamyl transferase (GGT), which were included to account for general inflammatory, metabolic, nutritional, and hepatic status. In subgroup analyses, participants were stratified by sex, age, race/ethnicity, education level, marital status, PIR, hypertension, alcohol consumption, smoking status, and diabetes status.

### 2.6. Statistical analysis

All analyses accounted for the complex NHANES sampling design using sampling weights, strata, and primary sampling units. Because six 2-year NHANES cycles from 2005 to 2006 to 2015 to 2016 were combined, Mobile Examination Center examination weights (WTMEC2YR) were divided by 6. The survey design was specified using the adjusted weights, SDMVSTRA, and SDMVPSU. Continuous variables were summarized as weighted mean ± standard error or weighted median with interquartile range, as appropriate. Categorical variables were presented as unweighted counts with weighted percentages. Between-group comparisons were performed using survey-weighted tests for continuous variables and Rao–Scott chi-square tests for categorical variables. Survey-weighted logistic regression models were used to evaluate the association between SIRI and clinically relevant depressive symptoms. SIRI was analyzed as both a continuous variable and quartiles, with the lowest quartile as the reference. Three models were constructed: Model 1 was unadjusted; Model 2 was adjusted for age, sex, and race/ethnicity; and Model 3 was further adjusted for education, marital status, hypertension, alcohol consumption, smoking status, BMI, systolic blood pressure, diastolic blood pressure, WBC, PLT, albumin, TG, alanine aminotransferase, aspartate aminotransferase, GGT, and PIR. RCS analysis and threshold effect analysis were performed to assess potential nonlinearity. ROC curve analysis was used to evaluate the incremental discriminatory value of SIRI. Subgroup analyses and interaction tests were conducted to examine effect modification. All analyses were performed using R software. A two-sided *P* value <.05 was considered statistically significant.

## 3. Results

### 3.1. Baseline characteristics of the study population

A total of 24,188 adults were included in this study, of whom 2055 had clinically relevant depressive symptoms based on the PHQ-9 cutoff. The median age of the overall population was 48 years (interquartile range: 34–63 years), and the median SIRI was 1.03 (0.70–1.51). Compared with participants without clinically relevant depressive symptoms, those with clinically relevant depressive symptoms had lower PIR levels and higher BMI, WBC, PLT, SIRI, fasting blood glucose, TG, and GGT levels. They were also more likely to be female, live alone, have a lower income, smoke, and have hypertension or diabetes. The SIRI level was higher in participants with clinically relevant depressive symptoms than in those without clinically relevant depressive symptoms (1.09 [0.73–1.63] vs 1.02 [0.70–1.50], *P* < .001) (Table 1).

**Table 1:** Survey-weighted baseline characteristics of participants according to depressive symptom status.

Variables	Total (n = 24188)	0 (n = 22133)	1 (n = 2055)	*P*
PIR, M (Q_1_, Q_3_)	2.16 (1.12, 4.12)	2.27 (1.19, 4.27)	1.22 (0.74, 2.23)	<.001
Age, M (Q_1_, Q_3_)	48.00 (34.00, 63.00)	48.00 (33.00, 63.00)	49.00 (36.00, 60.00)	.397
BMI, M (Q_1_, Q_3_)	28.00 (24.32, 32.50)	27.86 (24.30, 32.25)	29.60 (24.95, 34.78)	<.001
SBP, M (Q_1_, Q_3_)	120.67 (111.33, 132.67)	120.67 (111.33, 132.67)	119.33 (110.00, 132.67)	.011
DBP, M (Q_1_, Q_3_)	70.00 (62.67, 77.33)	70.00 (62.67, 77.33)	70.67 (63.33, 78.00)	.068
WBC, M (Q_1_, Q_3_)	6.90 (5.70, 8.40)	6.90 (5.70, 8.30)	7.40 (6.10, 9.00)	<.001
RBC, M (Q_1_, Q_3_)	4.65 (4.32, 5.00)	4.66 (4.33, 5.01)	4.58 (4.26, 4.92)	<.001
SIRI, M (Q_1_, Q_3_)	1.03 (0.70, 1.51)	1.02 (0.70, 1.50)	1.09 (0.73, 1.63)	<.001
Plt, M (Q_1_, Q_3_)	241.00 (204.00, 285.00)	241.00 (204.00, 284.00)	250.00 (209.00, 295.00)	<.001
TC, M (Q_1_, Q_3_)	191.00 (165.00, 220.00)	191.00 (165.00, 220.00)	193.00 (164.00, 223.00)	.468
FBG, M (Q_1_, Q_3_)	93.00 (85.00, 104.00)	93.00 (85.00, 104.00)	94.00 (86.00, 108.00)	<.001
TG, M (Q_1_, Q_3_)	122.00 (81.00, 189.00)	121.00 (80.00, 188.00)	137.00 (87.00, 210.00)	<.001
AST, M (Q_1_, Q_3_)	23.00 (20.00, 28.00)	23.00 (20.00, 28.00)	22.00 (19.00, 28.00)	<.001
ALT, M (Q_1_, Q_3_)	21.00 (16.00, 28.00)	21.00 (16.00, 28.00)	20.00 (16.00, 28.00)	.051
GGT, M (Q_1_, Q_3_)	20.00 (14.00, 30.00)	20.00 (14.00, 30.00)	22.00 (15.00, 36.00)	<.001
Education, n(%)				<.001
<High school	2325 (9.61)	2035 (9.19)	290 (14.11)	
>High school	12,896 (53.32)	12,064 (54.51)	832 (40.49)	
High school	8967 (37.07)	8034 (36.30)	933 (45.40)	
Marriage, n(%)				<.001
Live alone	9538 (39.43)	8430 (38.09)	1108 (53.92)	
Live with partner	14,650 (60.57)	13,703 (61.91)	947 (46.08)	
PIR, n(%)				<.001
<1.3	7488 (30.96)	6379 (28.82)	1109 (53.97)	
≥3.5	7625 (31.52)	7346 (33.19)	279 (13.58)	
1.3–3.49	9075 (37.52)	8408 (37.99)	667 (32.46)	
Sex, n(%)				<.001
Female	12,273 (50.74)	10,952 (49.48)	1321 (64.28)	
Male	11,915 (49.26)	11,181 (50.52)	734 (35.72)	
Age, n(%)				.030
<45	10,612 (43.87)	9757 (44.08)	855 (41.61)	
≥45	13,576 (56.13)	12,376 (55.92)	1200 (58.39)	
Race, n(%)				<.001
Mexican American	3826 (15.82)	3512 (15.87)	314 (15.28)	
Non-Hispanic Black	4849 (20.05)	4410 (19.92)	439 (21.36)	
Non-Hispanic White	11,110 (45.93)	10,201 (46.09)	909 (44.23)	
Other Hispanic	2213 (9.15)	1949 (8.81)	264 (12.85)	
Other Race	2190 (9.05)	2061 (9.31)	129 (6.28)	
Hypertation, n(%)				<.001
1	8380 (34.65)	7442 (33.62)	938 (45.64)	
2	15,808 (65.35)	14,691 (66.38)	1117 (54.36)	
Alcohol, n(%)				.753
1	17,454 (72.16)	15,965 (72.13)	1489 (72.46)	
2	6734 (27.84)	6168 (27.87)	566 (27.54)	
Smoking, n(%)				<.001
1	11,011 (45.52)	9766 (44.12)	1245 (60.58)	
2	13,177 (54.48)	12,367 (55.88)	810 (39.42)	
Diabetes, n(%)				<.001
1	2996 (12.39)	2596 (11.73)	400 (19.46)	
2	21,192 (87.61)	19,537 (88.27)	1655 (80.54)	

Depression was defined as PHQ-9 score >=10.

ALT = alanine aminotransferase, AST = aspartate aminotransferase, BMI = body mass index, DBP = diastolic blood pressure, FBG = fasting blood glucose, GGT = gamma-glutamyl transferase, PIR = poverty-to-income ratio, PLT = platelet count, RBC = red blood cell count, SBP = systolic blood pressure, SIRI = systemic inflammation response index, TC = total cholesterol, TG = triglycerides, WBC = white blood cell count.

### 3.2. Association between SIRI and depression risk

When SIRI was included as a continuous variable, higher SIRI was significantly associated with higher odds of clinically relevant depressive symptoms (Table 2). In the unadjusted model, each 1-unit increase in SIRI was associated with higher odds of clinically relevant depressive symptoms (OR = 1.08, 95% confidence interval [CI]: 1.03–1.13, *P* < .001). After adjustment for sex, race/ethnicity, and age, the association remained significant (OR = 1.12, 95% CI: 1.07–1.17, *P* < .001). After further adjustment for sociodemographic factors, lifestyle factors, clinical indicators, and laboratory markers, the positive association between SIRI and clinically relevant depressive symptoms persisted (OR = 1.07, 95% CI: 1.02–1.13, *P* = .004).

**Table 2:** Associations between SIRI and depression in logistic regression models.

Variable	Model 1 OR (95% CI)	*P*	Model 2 OR (95% CI)	*P*	Model 3 OR (95% CI)	*P*
SIRI, continuous	1.08 (1.03–1.13)	<.001	1.12 (1.07–1.17)	<.001	1.07 (1.02–1.13)	.004
SIRI quartiles						
Q1	1.00 (Reference)		1.00 (Reference)		1.00 (Reference)	
Q2	1.03 (0.88–1.14)	.980	1.05 (0.92–1.20)	.499	1.02 (0.89–1.17)	.823
Q3	1.09 (0.96–1.25)	.174	1.19 (1.04–1.36)	.011	1.10 (0.96–1.26)	.187
Q4	1.27 (1.12–1.45)	<.001	1.44 (1.26–1.65)	<.001	1.26 (1.10–1.44)	<.001
*P* for trend		<.001		<.001		<.001

Model 1: unadjusted, N = 24,145. Model 2: adjusted for sex, race/ethnicity, and age, N = 24,145. Model 3: adjusted for Model 2 plus education, marital status, hypertension, alcohol use, smoking status, BMI, SBP, DBP, WBC, PLT, Alb, TG, ALT, AST, GGT, and PIR, N = 24,145. P for trend was calculated by modeling SIRI quartiles as an ordinal variable in the corresponding logistic regression model.

CI = confidence interval, OR = odds ratio.

Quartile analysis showed that the association was mainly observed in the highest SIRI quartile. In the fully adjusted model, participants in Q4 had significantly higher odds of clinically relevant depressive symptoms than those in Q1 (OR = 1.26, 95% CI: 1.10–1.44, *P* < .001), whereas no significant differences were observed for Q2 or Q3 compared with Q1. These findings suggest that the association between SIRI and clinically relevant depressive symptoms may not be simply linear.

### 3.3. Nonlinear association and threshold effect analysis

**Figure 2.**
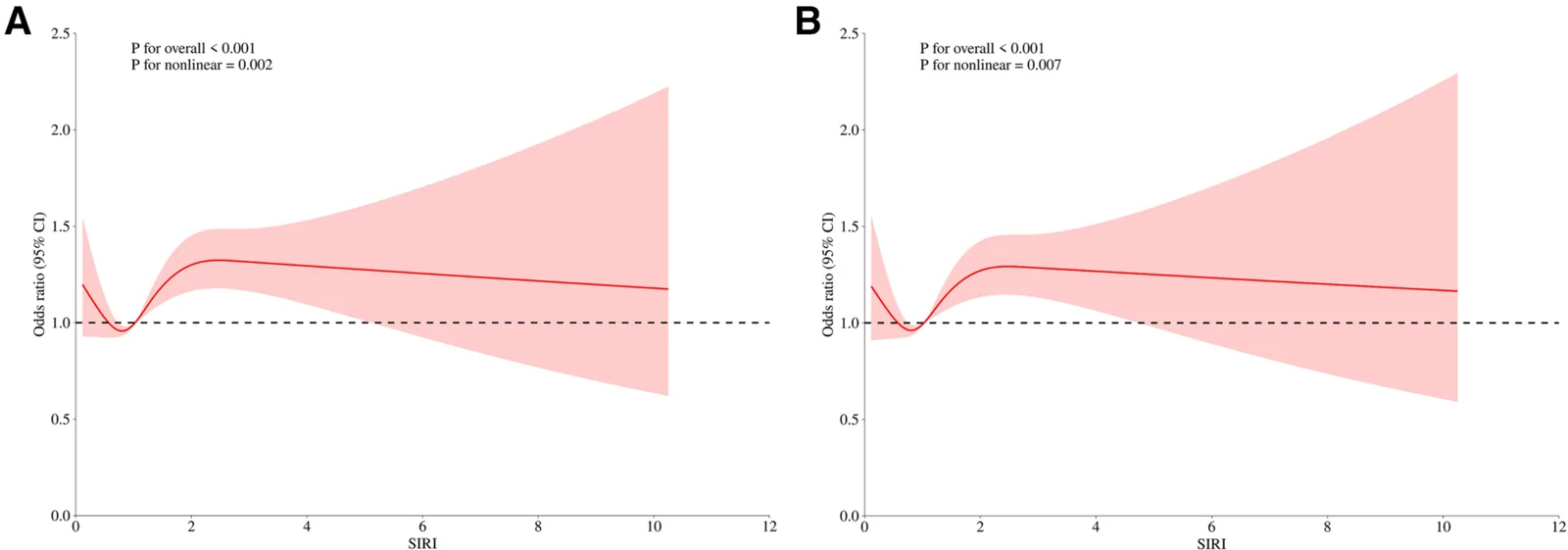
Restricted cubic spline analyses of the association between the systemic inflammation response index (SIRI) and depressive symptoms. (A) Unadjusted model. (B) Multivariable-adjusted model. The solid red line represents the odds ratio (OR), and the shaded area represents the 95% confidence interval (CI). The horizontal dashed line indicates OR = 1.

RCS analysis further demonstrated significant overall and nonlinear associations between SIRI and clinically relevant depressive symptoms (Fig. 2). In the unadjusted model, the overall association was significant (*P* for overall <.001), and a nonlinear relationship was observed (*P* for nonlinear =.002). In the multivariable-adjusted model, this nonlinear association remained significant (*P* for overall <.001; *P* for nonlinear = .007). According to the RCS curve, the odds of clinically relevant depressive symptoms increased as SIRI increased from approximately 0.70 to 1.50 (OR = 1.23, 95% CI: 1.12–1.36).

Threshold effect analysis showed that the inflection point was approximately 2.71 in the unadjusted model. When SIRI was <2.71, each 1-unit increase in SIRI was associated with higher odds of clinically relevant depressive symptoms (OR = 1.17, 95% CI: 1.07–1.27, *P* < .001); when SIRI was ≥2.71, the association was no longer significant. In the multivariable-adjusted model, the inflection point was approximately 2.58. When SIRI was <2.58, higher SIRI was significantly associated with higher odds of clinically relevant depressive symptoms (OR = 1.18, 95% CI: 1.08–1.29, *P* < .001), whereas the association tended to plateau when SIRI was ≥2.58 (OR = 0.99, 95% CI: 0.88–1.12, *P* = .875) (Supplementary Table 3, Supplemental Digital Content 3).

### 3.4. Subgroup analysis

**Figure 3.**
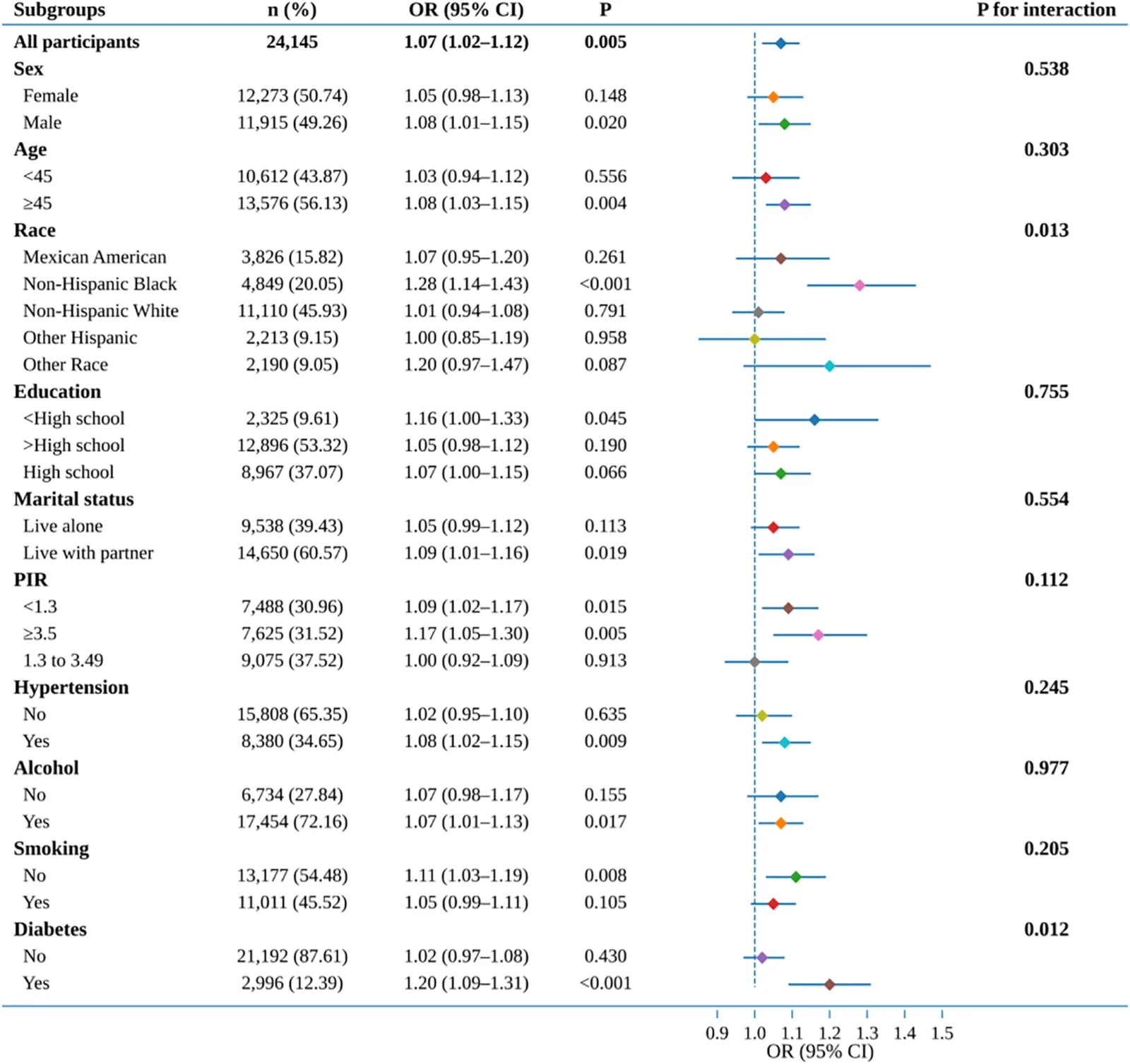
Subgroup analyses of the association between the systemic inflammation response index (SIRI) and depression. CI = confidence interval, SIRI = systemic inflammation response index.

Subgroup analyses were further performed to evaluate the association between SIRI and clinically relevant depressive symptoms. In the overall population, SIRI was significantly associated with clinically relevant depressive symptoms (Fig. 3), with an adjusted OR of 1.07 (95% CI: 1.02–1.12, *P* = .005). Further subgroup analyses showed that the interaction tests were not statistically significant across subgroups defined by education level, marital status, PIR, sex, age, hypertension, alcohol consumption, and smoking status, suggesting that these factors did not significantly modify the association between SIRI and clinically relevant depressive symptoms.

However, significant interactions were observed for race/ethnicity and diabetes status. In the race/ethnicity subgroup analysis, the *P* value for interaction was .013, and the association between SIRI and clinically relevant depressive symptoms was most pronounced among Non-Hispanic Black participants (OR = 1.28, 95% CI: 1.14–1.43, *P* < .001). In the diabetes subgroup analysis, the *P* value for interaction was .012. Among participants with diabetes, SIRI was significantly associated with clinically relevant depressive symptoms (OR = 1.20, 95% CI: 1.09–1.31, *P* < .001), whereas this association was not statistically significant among participants without diabetes (OR = 1.02, 95% CI: 0.97–1.08, *P* = .430). These findings suggest that the association between SIRI and clinically relevant depressive symptoms may differ by race/ethnicity and diabetes status.

### 3.5. ROC curve analysis

**Figure 4.**
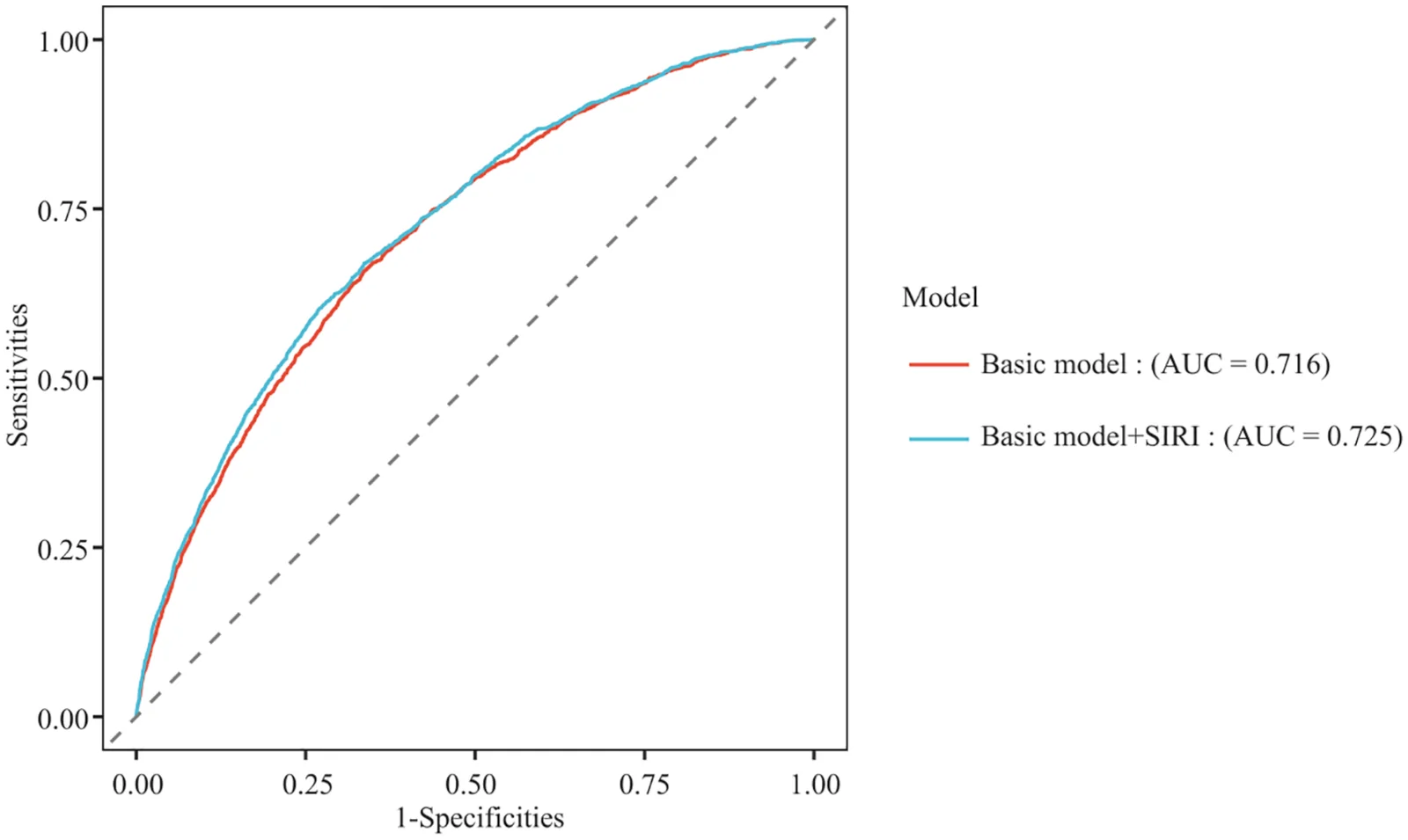
Receiver operating characteristic (ROC) curves for the basic model and the basic model plus systemic inflammation response index (SIRI) in identifying depression.

To evaluate the incremental discriminatory value of SIRI for identifying clinically relevant depressive symptoms, ROC curve analysis was performed (Fig. 4). The area under the curve of the basic model was 0.716 (95% CI: 0.705–0.728). After adding SIRI to the basic model, the area under the curve increased to 0.725 (95% CI: 0.714–0.736). The sensitivity increased from 0.659 to 0.670 after the addition of SIRI, whereas the specificity remained essentially unchanged (0.663 vs 0.664) (Supplementary Table 4, Supplemental Digital Content 4). These results suggest that SIRI slightly improved the discriminatory ability of the model for clinically relevant depressive symptoms, although the magnitude of improvement was limited. Therefore, SIRI may be more suitable as an auxiliary peripheral inflammatory reference marker rather than as a standalone screening or diagnostic tool.

## 4. Discussion

Based on adult participants included in NHANES 2005 to 2016, this study found that higher SIRI levels were associated with higher odds of clinically relevant depressive symptoms. Consistent positive associations were observed when SIRI was analyzed either as a continuous variable or as a quartile-based categorical variable. After full adjustment, each 1-unit increase in SIRI was associated with an approximately 7% higher risk of depression, and participants in the highest SIRI quartile had an approximately 26% higher risk of depression than those in the lowest quartile. RCS and threshold effect analyses further showed a nonlinear association between SIRI and depression, with the increased risk mainly observed within the low-to-moderate SIRI range and a plateau trend at higher SIRI levels. Unlike previous studies that mainly evaluated linear associations between inflammatory indices and depression, our study further identified a nonlinear association and a potential threshold effect of SIRI, providing additional insights into the complex relationship between systemic inflammation and depression.

The association between SIRI and depression may be related to the immune-inflammatory imbalance reflected by this index. SIRI integrates information from 3 types of peripheral immune cells, including neutrophils, monocytes, and lymphocytes, and may reflect the relative balance between innate immune activation and adaptive immune regulation. Elevated neutrophils and MCs generally indicate enhanced proinflammatory responses, whereas relatively reduced lymphocyte levels may suggest impaired immune regulation under chronic stress or inflammatory conditions. Therefore, elevated SIRI may represent an integrated inflammatory phenotype characterized by increased proinflammatory cell activation and relatively insufficient lymphocyte-related immune regulation.^[12]^ Notably, even after WBC and PLT were included in the fully adjusted model, the association between SIRI and depression remained significant, suggesting that SIRI may provide integrated immune-inflammatory information beyond single blood cell counts.

From a mechanistic perspective, activation of neutrophils and monocytes may promote the release of proinflammatory mediators such as IL-1β, IL-6, and TNF-α. These inflammatory signals can affect the central nervous system through the blood–brain barrier, vagal afferent signaling, or immune cell trafficking.^[13–15]^ In addition, inflammatory states may activate the tryptophan–kynurenine metabolic pathway, the hypothalamic–pituitary–adrenal axis, and microglial responses, thereby influencing serotonin synthesis, glutamate homeostasis, neuroplasticity, and emotion-regulation networks.^[16,17]^ Lymphopenia may also indicate impaired immune homeostasis and reduced anti-inflammatory regulatory capacity, allowing proinflammatory signaling to persist more easily.^[18]^ Thus, elevated SIRI may reflect a peripheral inflammatory–neuroimmune imbalance associated with depression.

This study further suggested a potential nonlinear threshold relationship between SIRI and depression. Compared with a simple linear model, this finding may better reflect the complex interaction between inflammation and mental health outcomes. Low-to-moderate increases in SIRI may indicate a gradual increase in chronic low-grade inflammation, a stage in which changes in inflammatory burden may be more closely related to depression risk. However, when SIRI reaches a higher level, its elevation may be more strongly influenced by acute infection, recent stress, medication use, or other somatic conditions, and may therefore no longer stably reflect a depression-related inflammatory phenotype. In addition, the relatively small sample size in the high-SIRI range may have contributed to unstable estimates and wider CIs. Therefore, the plateau trend between SIRI and depression should be interpreted with caution. This finding suggests that future studies evaluating inflammatory markers and mental health outcomes should not assume a simple linear relationship but should consider potential nonlinear dose–response patterns.

Subgroup analyses suggested that diabetes status and race/ethnicity may modify the association between SIRI and depression. The association was more pronounced among participants with diabetes, possibly because diabetes is characterized by chronic low-grade inflammation, insulin resistance, oxidative stress, and vascular dysfunction, all of which may contribute to depression.^[19]^ In this population, SIRI may not only reflect general peripheral inflammation but also capture a closer link between metabolic inflammation and mood-related symptoms. Differences by race/ethnicity should not be interpreted simply as biological differences; rather, they may reflect the combined influence of socioeconomic status, chronic stress exposure, healthcare access, comorbidity patterns, lifestyle factors, and baseline inflammatory status. Therefore, these subgroup findings should be regarded as exploratory and hypothesis-generating rather than definitive conclusions.

Although ROC analysis showed that adding SIRI to the basic model resulted in only a small improvement in discriminatory performance, this finding does not necessarily diminish the epidemiological relevance of SIRI. Depressive symptoms are multifactorial and are influenced by psychosocial, behavioral, metabolic, neuroendocrine, and inflammatory pathways; therefore, a single peripheral inflammatory marker would not be expected to provide strong discriminatory performance. The present findings suggest that SIRI should not be interpreted as a standalone screening or diagnostic marker for depressive symptoms. Rather, it may serve as an accessible peripheral inflammatory reference marker that provides auxiliary epidemiological information regarding inflammation-related depressive symptom burden.

This study has several limitations. First, the cross-sectional design precludes causal inference, and reverse causation cannot be excluded. Second, PHQ-9 ≥10 was used to identify clinically relevant depressive symptoms, but it cannot replace a clinical psychiatric diagnosis. Third, SIRI was calculated from a single blood test and may not reflect long-term inflammatory status. Fourth, despite adjustment for multiple demographic, socioeconomic, lifestyle, clinical, and laboratory covariates, residual confounding from unmeasured factors, such as medication use, acute or chronic inflammatory conditions, sleep disorders, psychosocial stress, and dietary factors, may remain. Fifth, participants with missing data were excluded, which may have introduced selection bias if excluded participants differed systematically from those included in the final analytic sample. Finally, although the addition of SIRI slightly improved model discrimination, the incremental discriminatory value was limited. Therefore, SIRI should be regarded as an auxiliary epidemiological inflammatory marker rather than a standalone screening or diagnostic tool.

## 5. Conclusion

Based on adult data from NHANES 2005 to 2016, this study found that higher SIRI levels were associated with higher odds of clinically relevant depressive symptoms, and this association showed a nonlinear pattern. The addition of SIRI to the basic model resulted in only a small improvement in discriminatory performance. These findings suggest that SIRI may provide auxiliary epidemiological information as an accessible peripheral inflammatory marker for understanding inflammation-related depressive symptom burden. However, given the modest effect size and limited incremental discriminatory value, SIRI should not be interpreted as a standalone screening or diagnostic tool for depressive symptoms. Further longitudinal and mechanistic studies are needed to clarify the causal relevance and clinical implications of this association.

## Acknowledgments

The authors thank the National Center for Health Statistics and all participants of the NHANES program for making these data publicly available.

## Author contributions

**Conceptualization:** Qiongqiong Zhan, Fei Ye.

**Data curation:** Qiongqiong Zhan, Yu Wu, Jiaojiao Sun, Yunan Liang.

**Formal analysis:** Qiongqiong Zhan.

**Investigation:** Qiongqiong Zhan, Yu Wu, Jiaojiao Sun.

**Methodology:** Qiongqiong Zhan, Yu Wu.

**Project administration:** Qiongqiong Zhan, Fei Ye.

**Resources:** Qiongqiong Zhan, Fei Ye.

**Software:** Qiongqiong Zhan, Yu Wu, Jiaojiao Sun.

**Validation:** Qiongqiong Zhan, Yu Wu.

**Visualization:** Qiongqiong Zhan, Yu Wu, Yunan Liang, Xinfeng Jiang, Fei Ye.

**Funding acquisition:** Fei Ye.

**Supervision:** Fei Ye.

**Writing – original draft:** Qiongqiong Zhan, Yu Wu, Jiaojiao Sun, Yunan Liang, Xinfeng Jiang, Fei Ye.

**Writing – review & editing:** Qiongqiong Zhan, Yu Wu, Jiaojiao Sun, Yunan Liang, Xinfeng Jiang, Fei Ye.








