# Supplementary material for: Association between systemic inflammation response index and depression in U.S. adults: A cross-sectional study from NHANES 2005–2016
Source: Medicine (Baltimore). 2026 Aug 21;105(34):e50379. doi: 10.1097/MD.0000000000050379 (PMC13502792; doi:10.1097/MD.0000000000050379)
Supplement: Supplementary file 1 [file medi-105-e50379-s001.docx]

**Supplementary Table 1. Participants excluded because of eligibility criteria or missing study variables**

| **Selection step** | **Denominator, n** | **Excluded, n** | **Excluded, %** | **Participants remaining, n** |
| --- | --- | --- | --- | --- |
| NHANES 2005–2016 participants | 60,935 | — | — | 60,935 |
| Age <20 years | 60,935 | 26,755 | 43.91 | 34,180 |
| Missing PHQ-9 depressive symptom assessment | 34,180 | 3,068 | 8.98 | 31,112 |
| Missing SIRI components | 31,112 | 1,751 | 5.63 | 29,361 |
| Missing major covariates | 29,361 | 3,231 | 11 | 26,130 |
| Missing laboratory variables | 26,130 | 1,942 | 7.43 | 24,188 |
| Final sample for baseline characteristic analysis | — | — | — | 24,188 |
| Final sample for multivariable regression analyses | 24,188 | 43 | 0.18 | 24,145 |

*Percentages were calculated using the number of participants remaining at the previous step as the denominator. SIRI was considered missing if any of its components, including neutrophil, monocyte, or lymphocyte count, was missing. PHQ-9, Patient Health Questionnaire-9; SIRI, systemic inflammation response index.*
