# Supplementary material for: Association between systemic inflammation response index and depression in U.S. adults: A cross-sectional study from NHANES 2005–2016
Source: Medicine (Baltimore). 2026 Aug 21;105(34):e50379. doi: 10.1097/MD.0000000000050379 (PMC13502792; doi:10.1097/MD.0000000000050379)
Supplement: Supplementary file 2 [file medi-105-e50379-s002.docx]

**Supplementary Table 2. PHQ-9 items and scoring criteria used for depression assessment in NHANES**

| **Section** | **NHANES variable / Score** | **Item or response option** | **Symptom domain / Description** |
| --- | --- | --- | --- |
| PHQ-9 item | DPQ010 | Little interest or pleasure in doing things | Core symptom A / Anhedonia |
| PHQ-9 item | DPQ020 | Feeling down, depressed, or hopeless | Core symptom B / Depressed mood |
| PHQ-9 item | DPQ030 | Trouble falling or staying asleep, or sleeping too much | Sleep disturbance |
| PHQ-9 item | DPQ040 | Feeling tired or having little energy | Fatigue / Loss of energy |
| PHQ-9 item | DPQ050 | Poor appetite or overeating | Appetite change |
| PHQ-9 item | DPQ060 | Feeling bad about yourself, or that you are a failure or have let yourself or your family down | Feelings of worthlessness or guilt |
| PHQ-9 item | DPQ070 | Trouble concentrating on things, such as reading the newspaper or watching television | Impaired concentration |
| PHQ-9 item | DPQ080 | Moving or speaking so slowly that other people could have noticed, or being so fidgety or restless that you have been moving around a lot more than usual | Psychomotor change |
| PHQ-9 item | DPQ090 | Thoughts that you would be better off dead or of hurting yourself in some way | Suicidal ideation |
| Scoring criterion | 0 | Not at all | Symptom absent during the past two weeks |
| Scoring criterion | 1 | Several days | Symptom present for several days during the past two weeks |
| Scoring criterion | 2 | More than half the days | Symptom present for more than half the days during the past two weeks |
| Scoring criterion | 3 | Nearly every day | Symptom present nearly every day during the past two weeks |

***Note:*** *Each PHQ-9 item is scored from 0 to 3 according to symptom frequency over the past two weeks. The total PHQ-9 score ranges from 0 to 27, with higher scores indicating more severe depressive symptoms. In this study, depression was defined as a PHQ-9 total score ≥10, whereas participants with a PHQ-9 total score <10 were classified as having no depression.*
