# Supplementary material for: Association between systemic inflammation response index and depression in U.S. adults: A cross-sectional study from NHANES 2005–2016
Source: Medicine (Baltimore). 2026 Aug 21;105(34):e50379. doi: 10.1097/MD.0000000000050379 (PMC13502792; doi:10.1097/MD.0000000000050379)
Supplement: Supplementary file 3 [file medi-105-e50379-s003.docx]

**Supplementary Table 3. Threshold analysis of the association between SIRI and depression.**

| **Model** | **Effect estimate (95% CI)** | **P** |
| --- | --- | --- |
| Unadjusted standard logistic regression | 1.08 (1.03-1.13) | <0.001 |
| Unadjusted two-piecewise model: inflection point | 2.71 |  |
| SIRI <2.71 | 1.17 (1.07-1.27) | <0.001 |
| SIRI >=2.71 | 0.97 (0.86-1.10) | 0.633 |
| P for likelihood ratio test |  | 0.030 |
| Fully adjusted standard logistic regression | 1.09 (1.04-1.14) | <0.001 |
| Fully adjusted two-piecewise model: inflection point | 2.58 |  |
| SIRI <2.58 | 1.18 (1.08-1.29) | <0.001 |
| SIRI >=2.58 | 0.99 (0.88-1.12) | 0.875 |
| P for likelihood ratio test |  | 0.039 |

*Fully adjusted model included sex, race/ethnicity, age, education, marital status, hypertension, alcohol use, smoking status, BMI, SBP, DBP, WBC, PLT, Alb, TG, ALT, AST, GGT, and PIR.*
