# Supplementary material for: Association between systemic inflammation response index and depression in U.S. adults: A cross-sectional study from NHANES 2005–2016
Source: Medicine (Baltimore). 2026 Aug 21;105(34):e50379. doi: 10.1097/MD.0000000000050379 (PMC13502792; doi:10.1097/MD.0000000000050379)
Supplement: Supplementary file 4 [file medi-105-e50379-s004.docx]

**Supplementary Table 4. ROC curve analysis for depression prediction models.**

| **Model** | **AUC** | **95% CI** | **Best threshold** | **Specificity** | **Sensitivity** |
| --- | --- | --- | --- | --- | --- |
| Basic model | 0.716 | 0.705-0.728 | 0.085 | 0.663 | 0.659 |
| Basic model + SIRI | 0.725 | 0.714-0.736 | 0.084 | 0.664 | 0.670 |

*AUC, area under the receiver operating characteristic curve; CI, confidence interval; SIRI, systemic inflammation response index.*
